# Two Cases of Compound Fracture of the Femur

**Published:** 1864-09

**Authors:** John Stainback Wilson

**Affiliations:** Surgeon Jackson Hospital.


**Art. VI.**—*Two Cases of Compound Fracture of the Femur.* Reported by John Stainback Wilson, Surgeon Jackson Hospital.

Case 1.—Thomas Lane, private 33d N. C., company "I," aged 45, farmer, previous health good, but of short stature and delicate appearance, and bilious temperament, was wounded on 12th May, 1864, by a minie ball, which entered the thigh antero-externally, five inches below anterior superior spinous process of ilium, and ranging obliquely upward and backward, made its exit just below the tuberosity of the ischium, fracturing femur. Admitted 18th May, with limb bound to a rough plank, having been thus transported from the battle-field, near Spottsylvania Court House. On 24th May the limb was placed on a double-inclined plane, with side pieces, and strips of coarse cloth extending from the lower edges of these, constituting thus the bottom of the box and the supporters of the thigh and leg. The foot was loosely bound to the foot-board, with the view of steadying the member, and guarding against any involuntary movements on the part of the patient, but with no intention of making extension or counter-extension.

No bandages or dressings of any kind were applied except a thin cloth, over the seat of injury, wet from time to time with cold water. During the whole progress of the case there was very little local inflammation; the discharge was slight, and the patient never had any fever or other constitutional disturbance.

July 6th.—Examined Lane to-day—union good—deformity not great, and the shortening is only two and a half inches. The patient shows me the ball, which is much battered; and there is a hardness beneath the skin on the anterior aspect of the thigh, which I feel assured is an encysted fragment of the wounding missile.

Case 2.—J. A. Dixon, private 10th Georgia battalion, company "B.," aged 26, farmer, previous health good, sanguine temperament, wounded 14th May, by a minie ball, which, entering the inner side of the thigh at the junction of the upper and middle thirds, and passing upward and outward, escaped five inches below anterior superior spine of ilium, fracturing femur.

Admitted 18th May, under much the same circumstances as those described in the case of Lane. Treatment the same, with like gratifying and truly remarkable exemption from local and constitutional excitement.

To-day, (July 6th,) I find the whole limb encased in a starch bandage, (immovable apparatus) applied by my successor, Dr. T. P. Perkins. This enables the patient to walk about on crutches, and altogether he is quite comfortable, with the exception of some pain from the pressure of the bandage, which may require re-adjustment. On examination, before the use of the starched apparatus, I found the union good, the deformity moderate, and the shortening *only one inch and a half* by careful measurement.

